# Host taxonomy and environment shapes insectivore viromes and viral spillover risks in Southwestern China

**DOI:** 10.1186/s40168-025-02115-9

**Published:** 2025-05-16

**Authors:** Ji-Hu Yang, Chun-Feng Luo, Rong Xiang, Jiu-Meng Min, Zong-Ti Shao, Yi-Lin Zhao, Lu Chen, Lin Huang, Yun Zhang, Shun-Shuai Liu, Yu-Qiong Li, En-Nian Pu, Wen-Qiang Shi, Hai-Feng Pan, Wei-Jun Chen, Chun-Hong Du, Jia-Fu Jiang

**Affiliations:** 1 State Key Laboratory of Pathogen and Biosecurity, Academy of Military Medical Sciences, Beijing, 100071 People’s Republic of China; 2 Yunnan Key Laboratory for Zoonosis Control and Prevention, Yunnan Institute for Endemic Diseases Control and Prevention, Dali, 671000 People’s Republic of China; 3 Huo-Yan Engineering Technology, BGI-Shenzhen, Shenzhen, 518083 People’s Republic of China; 4 Beijing Macro & Micro-Test Bio-Tech Co., Ltd, Beijing, 101300 People’s Republic of China; 5 School of Public Health, Anhui Medical University, Hefei, 230032 People’s Republic of China; 6 Zibo Center for Disease Control and Prevention, Zibo, 255020 People’s Republic of China; 7 College of Life Sciences, University of Chinese Academy of Sciences, Beijing, 100049 People’s Republic of China

**Keywords:** Emerging infectious diseases, Environment, Insectivore, Viral evolution, Virome

## Abstract

**Background:**

Zoonotic viruses originating from small mammals pose significant challenges to public health on a global scale. Insectivores, serving as natural reservoirs for a diverse array of zoonotic viruses, are known to carry a multitude of viral species. However, compared to the extensive research conducted on rodents (Rodentia) and bats (Chiroptera), the role of insectivores in harboring and transmitting unknown pathogens remains underexplored, which may lead to a severe underestimation of their contributions and impact to global public health.

**Results:**

This study employed a meta-transcriptomic approach to profile the viromes of 214 individual insectivores, encompassing 13 species from the families *Soricidae*, *Erinaceidae*, and *Talpidae*, collected across 12 counties in Yunnan Province, a recognized zoonotic hotspot. Based on virus reads, the analysis identified 42 viral families associated with vertebrates, highlighting significant virome diversity and host-specific viral tropisms among shrews, hedgehogs, and moles, along with notable geographic and environmental specificity of the viruses. Shrews exhibited greater viral richness and abundance compared to hedgehogs and moles, with variations influenced predominantly by host taxonomy, altitude, and geographic location. A total of 114 RNA-dependent RNA polymerase sequences were obtained, leading to the identification of 68 viruses, including 57 novel species. Instances of host jumping were observed in 11 viruses, with potential pathogenic viruses related to Mojiang paramyxovirus and members of the *Hantaviridae* family. Cross-species transmission was predominantly observed in viruses carried by shrews, while moles may play a pivotal role in facilitating viral transmission among insectivores.

**Conclusions:**

This study enhances the understanding of the high diversity of mammalian viruses among insectivores in a relatively confined region and underscores the associations between virome composition and related zoonotic risks, providing a foundation for proactive measures to prevent and control the spillover of emerging zoonotic pathogens and potential future outbreaks.

Video Abstract

**Supplementary Information:**

The online version contains supplementary material available at 10.1186/s40168-025-02115-9.

## Background

Emerging infectious diseases (EIDs) are predominantly zoonotic in origin, often arising from wild animals, and have increasingly gained attention in public health discussions [1]. With bats and pangolins identified as carriers of coronaviruses related to SARS-CoV-2, the potential spillover of diseases from animal hosts to humans has heightened public awareness and concern regarding viruses harbored by wildlife [2, 3]. There is a pressing need for comprehensive research into the viral spectrum present in wildlife hosts, including analyses of their prevalence, genetic diversity, and geographical distribution. Such studies are critical for enhancing the prevention and control of zoonotic EIDs [4].

Bats (Chiroptera) and rodents (Rodentia) stand as the most diverse groups among small mammals and act as key vectors in the transmission of zoonotic pathogens between humans and animals, playing a crucial role in the life cycle of vector-borne viruses. Insectivores, a term conventionally used to describe small mammals primarily feeding on insects, were historically grouped under the order Insectivora. In modern taxonomy, these mammals are now classified within the order Eulipotyphla, which includes families such as *Erinaceidae* (hedgehogs), *Talpidae* (moles), and *Soricidae* (shrews) [5]. In contrast to the extensive research on Rodentia and Chiroptera, the role of Eulipotyphla (insectivores) in pathogen carriage and transmission remains understudied, potentially leading to a substantial underestimation of their contributions and impact to public health and ecosystem dynamics. Eulipotyphla and Chiroptera belong to Laurasiatheria, and Eulipotyphla may have begun radiating earlier than Chiroptera and Rodentia [6]. In addition, arthropods are a major reservoir of viral genetic diversity and have likely been central to viral evolution [7]. It is speculated that the insectivores may harbor a more complex array of viruses that associated with rodents and bats and insect. Further investigation is essential to elucidate their epidemiological significance. Previous studies have extensively characterized the viromes of bats and rodents, systematic investigations into the virome diversity and transmission potential of insectivores remain notably limited [8–10]. Numerous pathogens originating from rodents and bats, such as *Coronaviruses*, *Hantaviruses*, *Babesia*, and *Anaplasma*, have been widely reported [11–17], and some of these pathogens have also been identified in insectivores [14–17]. Studies have demonstrated that viral diversity is influenced by host taxonomy, geographical location, and that landscape disturbances are positively correlated with mammalian viral diversity proliferation of viruses with multi-organ distribution in shrews, even within small sample sizes, compared to bats and rodents, which increases the likelihood of spillover events, as insectivores are frequently in close contact with humans [8, 18]. These findings underscore the urgency of analyzing the viral spectrum of insectivores, particularly shrews, to identify potential pathogenic viruses.

Yunnan Province, situated in Southwest China and bordering the Indo-Chinese peninsula, is known for its diverse eco-climatic zones and is recognized as a biodiversity hotspot, supporting more than half of China’s plant and animal species [19].Within China, the region hosts 59 species of shrews, 16 species of moles, and 7 species of hedgehogs, with at least 24, 9, and 2 species, respectively, found in Yunnan, highlighting its species diversity and its significance in studying the insectivore virome [20]. This area holds unique medical importance for evaluating potential spillover events of animal-borne zoonoses and the emergence of new pathogens [21–23]. Mammals that harbor more pathogens are more likely to be found in human-managed ecosystems, which increases the risk of zoonotic disease spillover from animals to humans [24]. This risk is particularly pronounced in Yunnan, which is one of the most popular tourist destinations in China. Therefore, investigating the diversity of insectivores and the viral spectrum they carry is of critical importance.

Meta-transcriptomics has been extensively utilized across various vertebrate species to identify both known and novel microbes, uncovering the evolutionary diversity, development, and origins of numerous zoonotic pathogens, despite significant challenges [22, 25, 26]. In this study, we collected insectivore samples and conducted meta-transcriptomic sequencing across 12 counties representing diverse habitats in Yunnan Province from 2015 to 2020. We specifically investigated the co-occurrence patterns among insectivores, viruses, and distinct geographic or ecological environments. Our findings revealed a high diversity of mammalian viruses among insectivores within a relatively confined area and demonstrated that both host species and environmental factors play a significant role in shaping the viromes of insectivores. These results provide essential groundwork for the proactive prevention and control of spillover events involving emerging zoonotic pathogens and potential future outbreaks.

## Methods

### Sample collection

Small wild mammals, including shrews, hedgehogs, and shrew moles, were captured using cages or traps baited with attractants, which were set out in the field at dawn and collected the following morning. The sample collection sites comprised 12 counties designated as plaque surveillance areas by the Yunnan Institute for Endemic Diseases Control and Prevention between 2015 and 2020. Ecological information, such as location, altitude, date, and habitat, was recorded. Captured small mammals were morphologically identified by experienced biologists and further confirmed through polymerase chain reaction amplification and then sequencing of the cytochrome b (*cytb*) and cytochrome *c* oxidase subunit I (COI) gene [27]. Aseptic dissections were then performed to obtain lung tissue samples. All collected samples were transported to the laboratory under cold-chain conditions and stored at – 80 °C until further processing.

### RNA extraction, library construction, and sequencing

Lung samples from each small mammal were homogenized in phosphate-buffered saline solution. The supernatant was filtered through 0.45 μm filter columns. Total RNA was extracted using a High Pure Viral RNA Kit (Roche Diagnostics, Catalog Number 11858882001). Prior to meta-transcriptomic sequencing, samples from the same insectivore species and location were pooled. The pooled samples were enriched using the Nucleic Acid Microbes Purification Kit (BGI PathoGenesis Pharmaceutical Technology) to ensure quality for sequencing. Reverse transcription and cDNA synthesis were performed using the PrimeScript Double Strand cDNA Synthesis Kit (TaKaRa Biotechnology, 6111A). Libraries were constructed using the QIAGEN QIAseq FX DNA Library Kit (QIAGEN, 180,477) and quantified with a Qubit 4.0 Fluorometer. Segment lengths were estimated using the Qsep-100 (Hangzhou Houze Biotechnology, Qseq100-a). Metagenomic high-throughput sequencing was carried out on the MGISEQ-2000 platform, with sterilized water used as a blank control to identify and eliminate potential sequence contamination. All samples and controls were sequenced on the same chip.

### Viral contig assembly and annotation

The raw sequencing data were processed using fastp (version 0.21.0) [28] to remove adaptor sequences and low-quality bases. The filtered reads were then de novo assembled into contigs using MEGAHIT (version 1.2.9) [29] with default parameters. These contigs were analyzed with DIAMOND blastx (version 0.9.21) [30] against the non-redundant (nr) protein database from GenBank (last updated January 2024), with an *E* value cut-off of 1 × 10^−5^. Contigs annotated under the kingdom “Viruses” were identified as potential viral contigs. These potential viral contigs were further subjected to blastn in BLAST software (v2.3.0 +) [31] search against non-redundant nucleotide (nt) databases from GenBank (last updated November 2020) to exclude host sequences, endogenous viral elements, and artificial vector sequences.

Following this, the viral contigs were manually validated and annotated using Geneious (v2024.0.5) [32], removing sequences that lacked annotation with viral RNA-dependent RNA polymerase (RdRp) or conserved replication-associated proteins, or that were shorter than half of the reference sequence. The species assignment of confirmed virome sequences was based on the classification criteria of each genus in the International Committee on Taxonomy of Viruses (ICTV), detailed in Table S4. For sequences lacking ICTV criteria, a 90% amino acid (aa) identity threshold was set for viral RdRp or conserved replication-associated proteins to classify them as novel virus species [10, 33].

### Metagenomic analysis

The identification of viral families was performed on the clean reads using Kraken2 [34]. The reads were quantified to determine the abundance of each viral family, and reads per million (RPM) were calculated based on the total number of reads in each library. To minimize false positives, a threshold of RPM ≥ 1 was set for detected viral reads, and only reads meeting this criterion were subjected to subsequent analysis.

Shannon–Wiener index calculations were performed using the known formula [35]. Principal component analysis (PCA) was conducted using the ade4 R package with default values using Euclidean distance, with all PCA plots, box plots, and heatmap figures generated utilizing the ggplot2 package in R version (4.0.3) [36, 37]. Differences between groups in PCA plots were assessed using pairwise permutational multivariate analysis of variance. The impacts of habitat and altitude on viral diversity were illustrated using principal coordinates analysis (PCoA) plot, and the significance was determined by adonis2 test, using ggplot2 package in R [37]. Comparisons of microbial diversity, microbe abundance, and gene expression levels between two groups were evaluated using the Wilcoxon rank-sum test, while comparisons between three groups were conducted using the Kruskal–Wallis test [38, 39].

### Phylogenetic analysis

Viral sequences were classified into major viral clades based on DIAMOND blastx results. To achieve accurate taxonomic assignments for newly identified viruses (both vertebrate and invertebrate-associated), we constructed phylogenetic trees for each viral clade using conserved viral proteins. Sequences were aligned with related viral sequences within the clade using MAFFT (v7.520) [40], and ambiguously aligned regions were trimmed with TrimAl (v1.4.rev15) [41]. Maximum likelihood phylogenetic trees were estimated from the aa alignments using IQ-TREE (v2.2.0.3) [42], with the best-fit substitution model determined by the “-m MFP” setting and ultrafast bootstrap approximation with 1000 replicates. All trees were visualized using FigTree (v1.4.4).

### Host-virus correlation analysis

Host information of known viruses was acquired from National Center for Biotechnology Information (NCBI) nt database up to August 12, 2024. A virus with two or more host species, genus or family was identified as of cross-species, cross-genus or cross-family transmission risks, respectively. A virus with two or more host order was identified as of spillover risks [43]. The correlation network among hosts and viruses was generating by Cytoscape (v3.10.1) software [44] with default values. Node sizes were used to distinguish host and virus. Node colors were used to distinguish host types and virus types. Node border colors were used to distinguish transmission patterns.

## Results

### Sample information of wild insectivore mammals

**Fig. 1:**
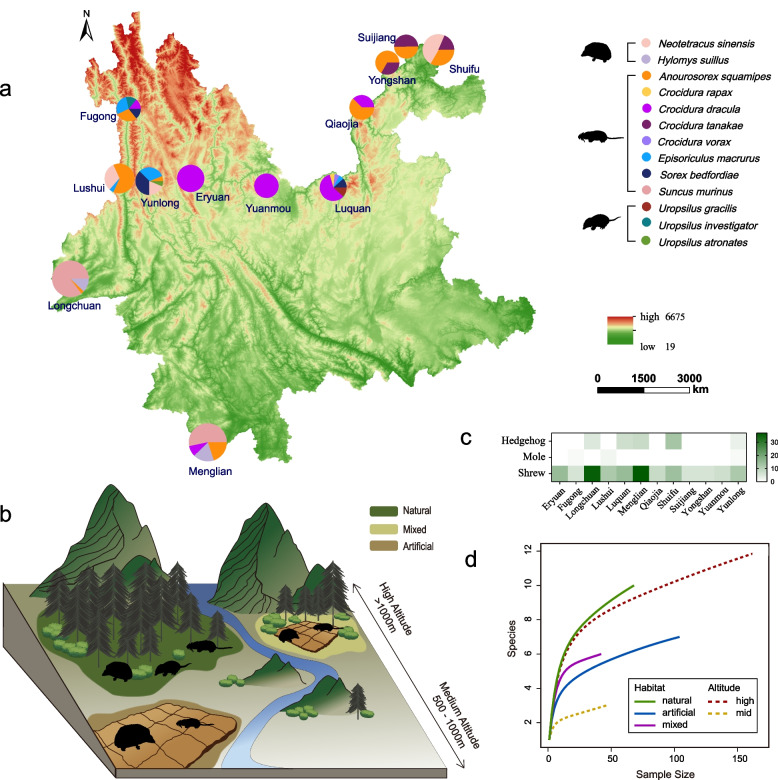
Sample locations, landscapes, and wild insectivore compositions. **a** Distribution of mammals sampled in different counties in Yunnan Province, with different pie charts representing various mammal species and their percentages in each county, and the diameter of each pie chart indicating the number of samples collected. Altitude of each location is illustrated from lowest (green) to highest (red). **b** Landscape illustration depicting the habitats and altitudes of the collected insectivores. **c** Heatmap showing the number of samples collected from three insectivore groups in each county. **d** Rarefaction curve illustrating the insectivore sample sizes across different habitats and altitudes

From November 2015 to December 2020, field surveys of insectivores were conducted across 12 counties in Yunnan Province, China (Fig. 1a and Table S1). The sampling sites encompassed various habitats, including natural environments (broad-leaved forests, coniferous forests, and bushlands), artificial settings (cultivated lands and residential areas), and mixed habitats, with altitudes ranging from 500 to 3561 m (Fig. 1b).

A total of 214 individual insectivores belonging to 13 species were captured, including two species of hedgehogs, 3 species of shrew mole, and 8 species of shrews (Fig. 1c and Table S1). The majority of the samples (174/214, 81.31%) were shrews, while moles represented a small proportion of the sample size (4/214, 1.87%). The most frequently captured insectivore was *Suncus murinus* (59/214, 27.57%), while *Anourosorex squamipes* (47/214, 21.96%) was the most widely distributed species, identified in 9 of the sampled counties. To assess whether the sampled insectivores accurately reflect their true richness across different habitats and altitudes, a rarefaction curve was generated, showing diminishing trends across these variables (Fig. 1d). This suggests that the insectivores captured in this study generally represent their true abundance across various habitats and altitudes.

### Meta-transcriptomic analysis and insectivore virome

A total of 214 individual insectivore RNA samples were pooled into 68 groups (Table S2) for analysis. Sequencing generated a total of 2.585 terabases of paired-end reads, which comprised 18,745,507,832 high-quality reads, each with a length of 150 bp. These reads were subsequently used for viral genome assembly and identification. To identify viral contigs, sequences annotated under the taxonomic kingdom “Viruses” were retained as candidate viral contigs, while non-viral sequences were discarded. This filtering process yielded 17,344 contigs (≥ 200 bp) that exhibited the best matches to viral sequences in the nt database. Notably, the number of virus-associated contigs varied significantly across pools, ranging from 3 to 360, reflecting the diverse viral composition within the samples.

**Fig. 2:**
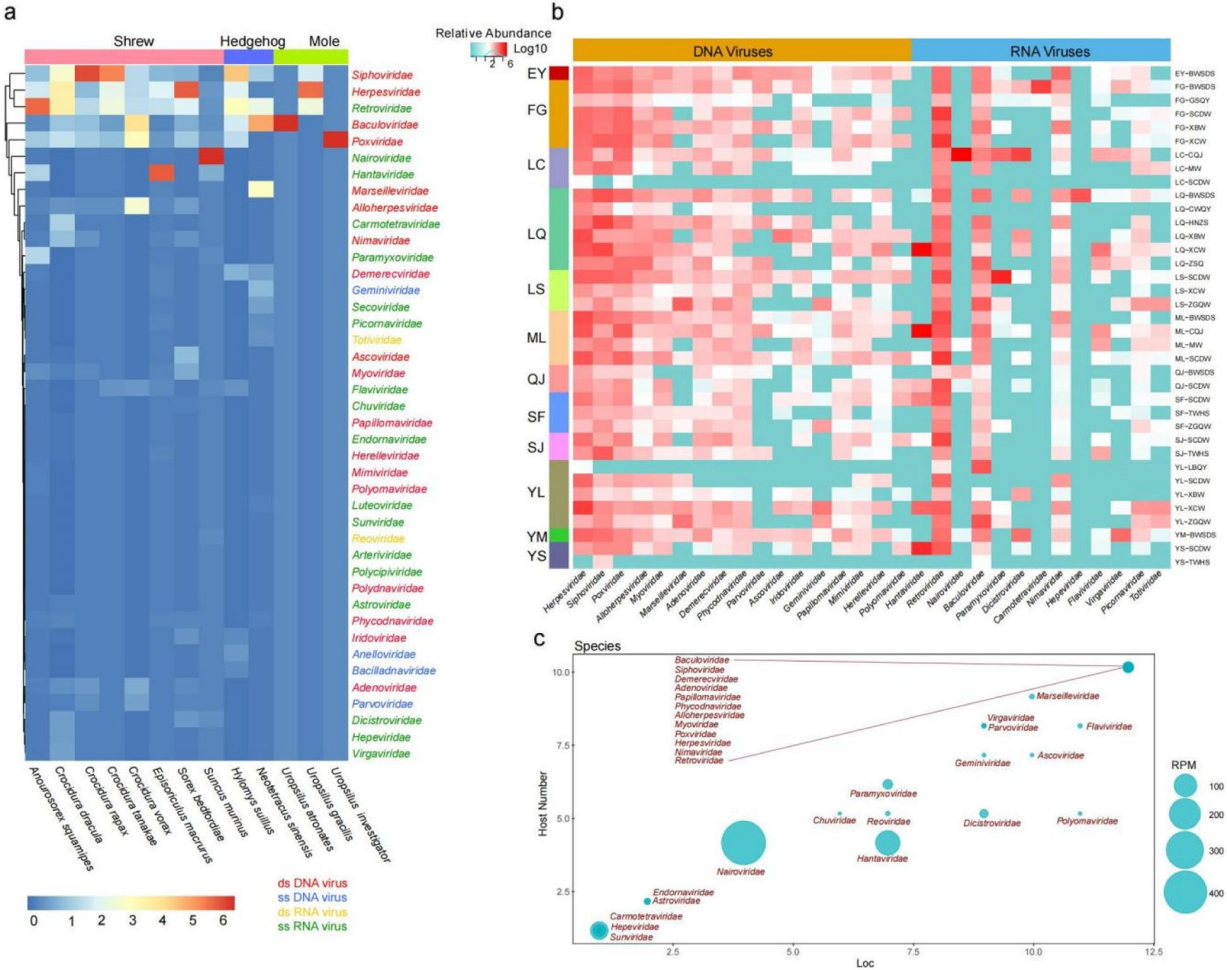
Characterization of insectivore viromes. **a** Heatmap based on the relative abundance of different host species, with Euclidean distance calculated and clustering performed using the complete method; insectivore types are displayed in various colors at the top. **b** Heatmap based on the relative abundance of the 30 most abundant viral families in each pooled sample, with sample locations listed on the left and species on the right; viral family names are at the bottom, and boxes colored from green to red represent the normalized viral reads for each sample. Abbreviations of locations and host species can be seen in Table S2. **c** Prevalence of viral families in host species and locations, where the x-axis represents the number of counties and the y-axis represents the number of host species in which a certain virus was found; the size of the circle denotes the reads per million for each viral family

The sequencing reads covered a broad spectrum of DNA and RNA virus groups. Virus-associated reads were classified into 42 families, encompassing double-stranded (ds) DNA viruses, dsRNA viruses, retrotranscribing viruses, single-stranded (ss) DNA viruses, and ssRNA viruses. Many of the sequence reads related to mammalian viruses exhibited low nt and aa sequence identity with known viruses (Fig. 2a).

### Viral abundance and richness

*Baculoviridae*,* Herpesviridae*, and *Poxviridae* were found to be abundant across all three insectivore families. In contrast, *Hantaviridae* and *Nairoviridae* were more prevalent in shrews, with lower relative abundances in hedgehogs and moles, indicating a broad host distribution for these families. Specifically, *Baculoviridae* was more prevalent in moles, while *Marseilleviridae* was more common in hedgehogs, suggesting a host preference among different viral families (Fig. 2a). On the other hand, significant pathogenic viral families such as *Siphoviridae*, *Retroviridae*, *Hantaviridae*, *Nairoviridae*, and *Alloherpesviridae* were predominantly associated with shrews, suggesting that shrews may harbor a higher number of pathogenic viruses (Fig. 2a).

The relative abundances of the 30 most prevalent viral families in pooled samples from different species and locations are shown in Fig. 2b. Reads from the families *Herpesviridae*, *Siphoviridae*, *Poxviridae*, *Alloherpesviridae*, *Retoviridae*,* Baculoviridae*, *Adenoviridae*, and *Nimaviridae* were detected in most insectivores and counties. In contrast, the families *Hepeviridae* was found in fewer insectivore species and counties, which may suggest more limited natural reservoirs and transmission scopes, potentially posing a relatively lower public health risk. Notably, while *Hantaviridae* and *Nairoviridae* were present in a moderate number of species and counties, their viral abundance was distinctly higher (Fig. 2c), which implies a greater likelihood of potential to cause outbreaks and threats to human health, calling for enhanced surveillance and proactive prevention strategies.

### Factors shaping the insectivore virome

**Fig. 3:**
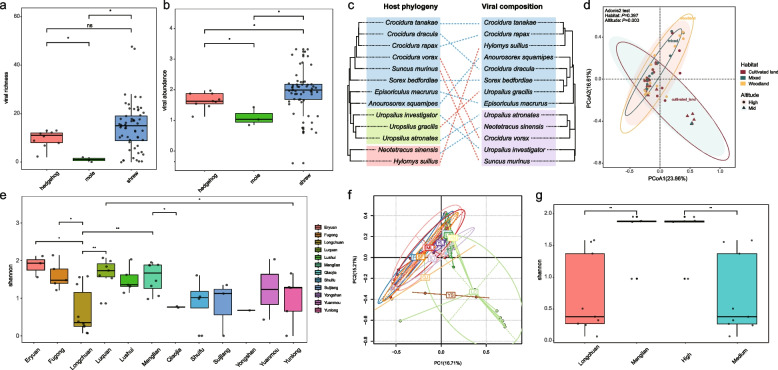
Environmental and host factors affecting viral diversity and distribution. **a** Difference in viral richness among different insectivores, with “*” indicating *P* < 0.05, “**”’ indicating *P* < 0.01, and “ns” indicating not significant. **b** Difference in viral abundance among different insectivores. **c** Relationships between virome composition and host phylogeny of different insectivore species based on the RPM table. **d** Difference in viral composition of shrews across different habitats and locations based on the RPM table. **e** and **f** Differences in Shannon index of shrews at different locations. **g** Difference in Shannon index of *S*. *murinus* at different locations and altitudes

Further analysis reveals significant variation in viral richness and abundance among different insectivore types, indicating that shrews harbor more viruses both in terms of number and abundance (Fig. 3a, b). The viral compositions of shrews form a separate clades with those of hedgehogs and shrew moles. Specifically, the viral composition profiles of *Crocidura* *vorax* (shrew), *S*. *murinus* (shrew), *U*. *gracilis* (shrew mole), and *H*. *suillus* (hedgehog) differs from the phylogenetic relationship characteristic of their respective host species, suggesting potential host shift. Meanwhile, the similarities with the genera *Sorex*, *Episoriculus*, and *Neotetracus* suggest a scenario of co-evolution (Fig. 3c). These findings warrant serious consideration and further investigation.

The viral composition in shrews varies significantly with altitude. According to the PCoA plot, shrews at high and medium altitudes exhibit distinct viromes (adonis2 test, *P* < 0.05). Specifically, the virome of shrews at medium altitude is more diverse compared to those at high altitude. In contrast, no significant differences in viral composition were observed among different habitats of insectivore hosts (adonis2 test, *P* > 0.05) (Fig. 3d). Additionally, shrews from different counties show significant variability, with those from Eryuan, Menglian, and Luquan exhibiting greater virome diversity (Fig. 3e). Notably, the viral diversity at Yongshan and Longchuan clusters is distinct from other counties (Fig. 3f). Among the shrews studied in this paper, *S*. *murinus* have the largest sample sizes and were further analyzed to determine factors affecting viral composition. *S*. *murinus* showed significant influence from location and altitude on viral composition, with statistical significance (Fig. 3g). It reveals the impact of altitude and region on shrew viromes, offering insights into viral adaptation, ecosystem–virus interactions, and enriches the understanding of ecological niches, highlighting the complex relationships among environment, host, and virus.

### Phylogeny of insectivore viruses

We identified and assembled 114 viral RdRp sequences representing 68 viral species from 11 viral families with pathogenic potential to humans based on meta-transcriptomic data, of which 57 were novel viruses and 11 were known viruses. Specifically, 101 sequences were obtained from shrew hosts, 7 from mole hosts, and 6 from hedgehog hosts. The viruses discovered in this study exhibit high diversity and establish unique evolutionary relationships across 12 insectivore species, with no viral species identified in *U. atronates* (Table S3). The detailed genome structure of 57 new viral species was individually verified (Fig. S2).

**Fig. 4:**
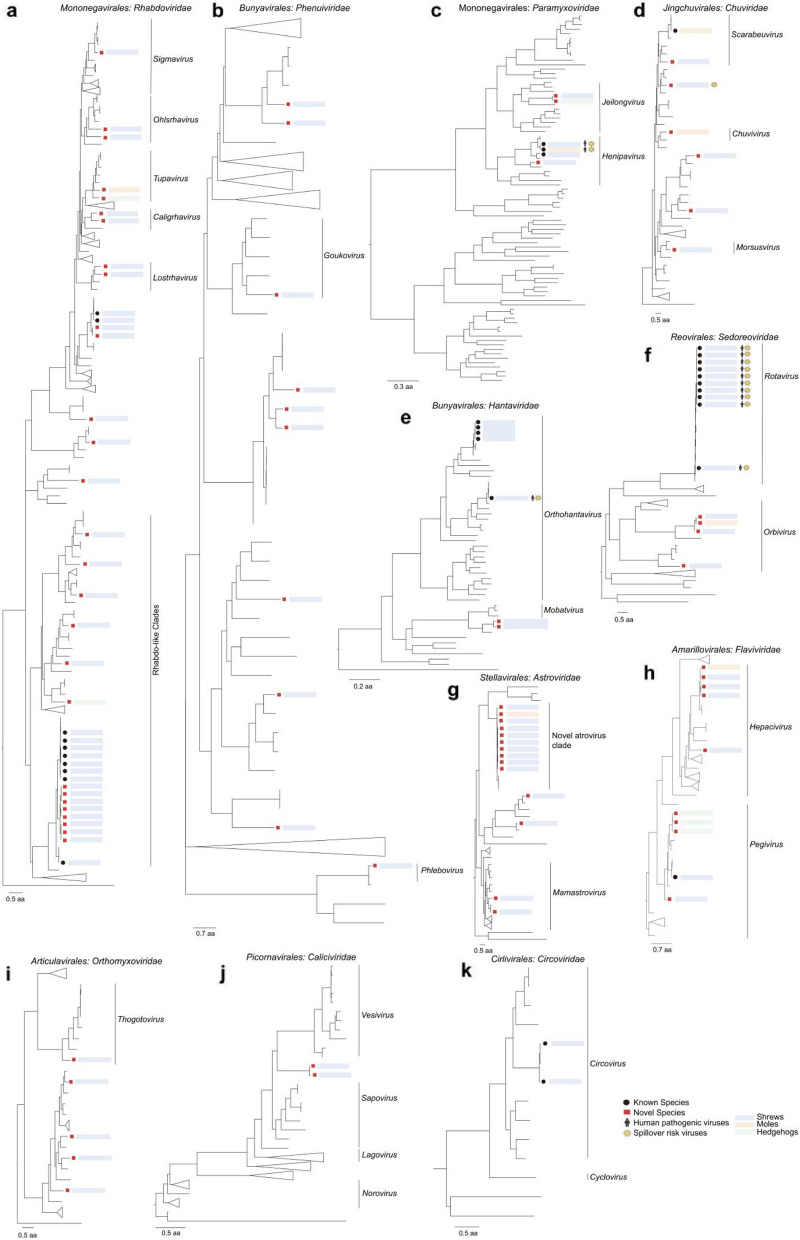
Phylogenetic diversity of vertebrate- and invertebrate-associated viruses. Phylogenetic trees were estimated based on amino acid sequences of the RdRp protein for RNA viruses and the replicase protein for DNA viruses. Phylogenetic inference was performed using the maximum likelihood (ML) method with 1000 bootstrap replicates. Branch lengths are indicated by the scale bar. Viruses identified in this study are color-marked according to their hosts, with blue, red, and yellow representing shrews, moles, and hedgehogs, respectively. Novel viruses discovered in this study are labeled with black rectangles, while viruses of known species are labeled with black circles. Human pathogenic viruses are indicated with black figures, and spillover-risk viruses are marked with yellow polygons. See also Fig. S1. Abbreviations of locations and host species can be seen in Table S2

Phylogenetic analyses of the viruses identified here revealed that insectivore harbored a high diversity of vertebrate and invertebrate associated viruses, including those of evolutionary significance (Fig. 4, Fig. S1). In the family *Rhabdoviridae*, there had the largest number of newly identified viruses (24/57) across all three insectivore families, underscoring the role of insectivores as significant hosts in viral evolution (Fig. 4a). Among of them, three novel species of shrew rhabdovirus, provisionally named Shrew rhabdovirus BIME1, BIME2, and BIME3, located between the clades of Wufeng shrew rhabdovirus 2 and 3 (Fig. S1a), with less than 70% aa identity compared to these known viruses. Seven viruses within the family *Chuviridae*, two identified in moles and five in shrews, formed separate clades (Fig. 4d, Fig. S1d), indicating independent evolution with hosts. In the family *Astroviridae*, a novel virus with diverse strains demonstrated 84.3% to 88.4% aa (Table S3) identity with the closest known astrovirus (Wenzhou rodent astrovirus 1) and formed a sister clade with it (Fig. S4, Fig. 4g). Two genera within the family *Sedoreoviridae* displayed different geographical distributions. Among them, *Rotavirus* was found in Menglian and Longchuan, and a strain of Rotavirus A from *S*. *murinus* exhibited a high aa identity (97.9%) with the Rotavirus A that infects humans (Table S3), suggesting a close evolutionary relationship and a potential risk of zoonotic infection (Fig. 4f).

Within the *Hantaviridae* family, three strains of Cao Bang viruses, one Orthohantavirus seoulense (SEOV), and one novel hantaviruses (provisionally named Shrew hantavirus BIME1) were identified (Fig. 0.4e, Fig. S1e). This newly discovered hantavirus is closely related to a hantavirus previously detected in *Chodsigoa hypsibia* shrews in China, sharing an amino acid identity of 88.26–88.59% (Table S3). In the family *Paramyxovidae*, two sequences with a high degree of similarity (93.02% aa identity) to Mojiang paramyxovirus (MojV) were found within the genus Henipavirus. Two novel viruses within the genus Jeilongvirus exhibited an amino acid identity of 65.87% with the closest known viru*s,* Belerina virus, which was identified in Belgian hedgehogs (Fig. 4c, Table S3). In addition, there were many known or novel viruses that belong to the family *Phenuiviridae* (Fig. 4b), *Flaviviridae*, *Orthomyxoviridae, Caliciviridae*, and *Circroviridae* (Fig. 4h–j), respectively.

### Transmission of insectivore virome

**Fig. 5:**
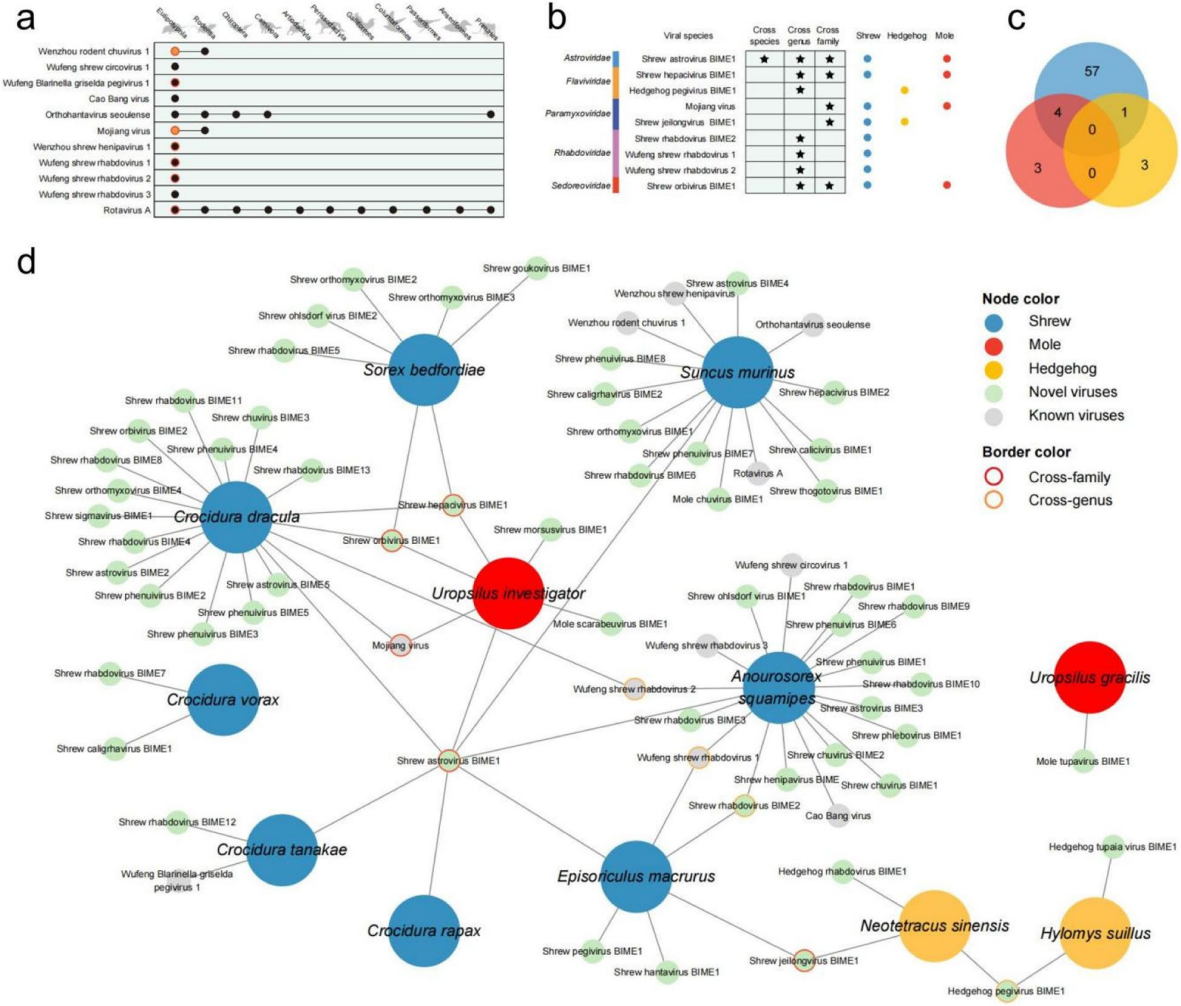
Cross-species transmission of viruses. **a** Overview of transmission across different host orders of known viruses. Black circles represent host orders of various viruses discovered by other studies. Orange circles represent host orders discovered by our study. Red rings denote new hosts discovered in our study. **b** Overview of transmission across host species, genus, and family for new viruses. **c** Venn diagrams illustrating the number of viruses in and shared by shrews, hedgehogs, and moles. **d** Host-virus correlation network. Node colors represent insectivore species and virus species, with node border colors indicating viruses that transmit among insectivores

Among the examined shrews, most of the 68 viral species from 11 families identified in 12 species of insectivores were found to be exclusively present in a single host species (59/68, 86.76%). However, some known viral species were either first recorded in insectivore species or showed expanded host ranges compared to previous records from NCBI nucleotide database. For instance, SEOV and MojV, previously known to infect rodents, were also detected in *S*. *murinus*, *Uropsilus* *investigator*, and *Crocidura* *dracula* in this study (Fig. 5a). It indicates that MojV pose public health risks and a high spillover risk due to their wide host range as well as high matched known zoonotic. We also discovered *S. murinus* as a host for Rotavirus A, and that Wenzhou rodent chuvirus 1 and MojV have cross-order transmission capabilities. New insectivore hosts were also identified for Wufeng Blarinella griselda pegivirus 1, Wenzhou shrew henipavirus 1, Wufeng shrew rhabdovirus 1, and Wufeng shrew rhabdovirus 2. Host jumping were observed in a total of 11 viruses (Fig. 5a).

Notably, 9 newly identified viruses were found across different insectivore hosts, indicating potential for cross-species, genus, or family transmission (Fig. 5b). Most of them were identified in shrews, with cross-family transmission primarily observed between shrews and moles (Fig. 5c). *A*. *squamipes* harbors the most diverse array of viral species, followed by *S*. *murinus* and *Cr*. *dracula.* In contrast, *Cr*. *vorax* (shrew) and *U*. *gracilis* (mole) host viruses that appear to be relatively independent of those found in other insectivore species (Fig. 5d). It is noteworthy that most viral transmissions were observed across genera rather than within species or families. Cross-genus transmission of viruses is predominantly identified in *A*. *squamipes*, with shared occurrences among *Episoriculus* *macrurus* and *Cr. dracula*, although no evidence of cross-genus transmission was observed specifically in this species of shrews. Cross-family transmission was found between shrews and moles, as well as between shrews and hedgehogs, while no viral interactions were noted between moles and hedgehogs. Notably, *U*. *investigator* (mole) appears to play a role in the cross-genus transmission of viruses between different shrew genera (Fig. 5d).

Viral interactions between shrews and hedgehogs were observed, particularly involving the genus *Episoriculus* in shrews and *Neotetracus* in hedgehogs. Among these interactions, a novel virus, Shrew Jeilongvirus BIME1 from the family *Paramyxoviridae*, was identified. Additionally, a novel virus named Shrew astrovirus BIME1 was detected across multiple insectivore species, including six shrew species and one mole species (Fig. 5d).

## Discussion

Our study offers a comprehensive analysis of viruses in shrews, hedgehogs, and moles, mapping the richness and diversity of insectivore viruses. This further supports the finding that shrews harbor a high variety of mammalian viruses [18]. While other studies have focused on shrews across larger geographical areas with limited species, our study includes three families of insectivores spanning 13 species and multiple locations in Yunnan Province [9, 18]. This broader sampling provides a more accurate representation of the insectivore virome and allows for a comparative analysis of viral composition across different insectivore families.

Viruses carried by rodents and bats have drawn global attention and have been extensively studied. However, recent researches have identified more insectivore-borne viruses, previously thought to be exclusive to rodents and bats, such as coronaviruses, severe fever with thrombocytopenia syndrome virus, and hantaviruses, in shrews [14, 45–47]. This raises important questions about the role of insectivores as reservoir hosts in the co-evolution of viruses and their potential for shedding viruses and spillover to humans. The discovery of Langya virus in febrile patients in eastern China, which was also found in shrews from the same area, underscores these concerns [48]. Our study further expands on this by confirming a broader range and diversity of viruses in shrews and identifying viruses in moles and hedgehogs, which are in the same order as shrews. This highlights the necessity of monitoring the virome of insectivores to better understand relevant pathogens and prevent zoonotic spillovers.

Many studies have focused on respiratory and anal tract samples, which are easily acquired through noninvasive methods. While these samples can partially represent the shedding of viruses from the host, they do not definitively distinguish between viruses originating from the environment versus those harbored by the host. To address this, studying the virome in host organs or tissues—referred to as the “core virome”—provides a more accurate representation of viral infections in reservoir hosts. This approach helps to differentiate between viruses that are directly from host infections and those that are indirectly acquired from the environment.

For example, Shi et al. examined core viromes in organs of amphibians, reptiles, and fishes, identifying distantly related vertebrate viruses and elucidating the evolutionary history of vertebrate RNA viruses [49]. However, large-scale monitoring of core viromes in insectivore organs and tissues remains limited. Our study addresses this gap by investigating the core viromes of insectivores, confirming that viruses can infect and be carried by these animals, and highlighting their crucial role in virus evolution and shedding.

Among the 68 viruses identified in our study, an impressive 86.76% (59/68) were novel, highlighting the ongoing need for meta-transcriptomic research to map the insectivore virome and discover new viruses. Vector-borne or invertebrate-associated viruses, such as those transmitted by mosquitoes and ticks, are of significant public health concern [50–52]. Insectivores, which feed on insects, are at a higher risk of virus spillover from insects to vertebrate animals, given the diverse array of known viruses from families such as *Flaviridae*, *Dicistroviridae*, *Permutotetraviridae*, and *Spinareoviridae* [18]. Our study also identified several novel viruses in the *Rhabdoviridae* and *Chuviridae* families, which are closely related to invertebrate-associated viruses. Understanding the relationship between vector-borne viruses and insectivores may reveal new pathways for virus transmission from insects to insectivores and ultimately to humans.

Ecological factors such as host taxonomy, location, altitude, habitat, and human interference play a crucial role in shaping the viral composition of hosts. The model developed by Chen et al. demonstrated that factors such as host order, sample size, and habitat significantly influence the virome of small mammals, and these variations can also occur in different organs [18, 53]. Our study, conducted over a broad geographical range with a diverse set of insectivore species, confirmed that host taxonomy, altitude, and geographical distribution significantly impact the viral composition in lung tissues of hosts. Research on other vectors, such as ticks, has also indicated that altitude affects virus evolution [54]. Although our study found an influence of altitude on viral composition, the mechanisms underlying this effect warrant further investigation. Our result shows that altitude can affect the virome of *S*. *murinus*, which aligns with the finding by Chen et al. [18]*.* Additionally, another study found that rodents in habitats with high anthropogenic disturbance exhibited the highest viral diversity [21]. This discrepancy may be attributed to our classification of habitats based on human interference, where cultivated lands are considered high interference habitats, whereas woodlands are considered low interference.

Our study reveals a high abundance of viruses from the families *Hantaviridae* and *Nairoviridae*, which warrants increased attention due to the significant public health implications associated with pathogens from these families [55–57]. Previous research has identified various genera and species of *Hantaviridae* in shrews and other insectivores across different provinces in China. For instance, *Imjin* virus and *Thottapalayam* virus were reported in Zhejiang, Kenkeme, and Khabarovsk virus in Heilongjiang, and Lianghe virus in Yunnan [46, 58, 59]. Our study similarly identified two hantavirus species in Yunnan province. The Cao Bang virus was found in *A*. *squamipes*, consistent with findings from Vietnam, Guizhou, and Taiwan, highlighting the host specificity of Cao Bang virus [58]. Conversely, SEOV traditionally associated with rodents such as *Rattus norvegicus* and *R*. *rattus*, was detected in *S*. *murinus* in our study, extending the known host range of this virus [60]. Furthermore, as SEOV is confirmed to be pathogenic to humans, our findings underscore its potential for cross-order transmission [61].

Notably, in addition to SEOV in *Hantaviridae*, MojV from *Paramyxoviridae* was also detected in our study. This virus was initially identified in rats, and its potential pathogenicity has been further confirmed through in vitro cellular experiments [62, 63]. *Paramyxoviridae* viruses have been documented in rodents, bats, and shrews, with a notable positivity rate in shrews reaching 49% in Belgium [64]. The recent identification of Langya henipavirus, which is phylogenetically close to MojV, in patients experiencing fever underscores the heightened concern regarding these viruses [48]. In our study, MojV was found in not only a species of shrews (*Cr*. *dracula*) but also a mole species (*U*. *investigator*), indicating a high risk of spillover. We also identified other *Paramyxoviridae* viruses, including a novel Jeilongvirus, which is closely related to *Belerina* virus previously detected in Belgian hedgehogs and bats in China [65, 66]. This suggests that shrews may harbor viruses from the *Paramyxoviridae* family. Therefore, further investigation into potential mutations in these hosts and a thorough evaluation of spillover risks are warranted.

Viruses capable of infecting hosts from multiple orders are considered at higher risk for mutations and spillovers [43]. For instance, coronaviruses have been documented to jump between species, such as from bats to hedgehogs and other animals, posing significant risks to humans and domestic animals [47]. Previous research has confirmed that rodents are at a higher risk of harboring cross-species transmitted viruses compared to bats and shrews [18].

Our study provides further insight into virus transmission patterns within the order Eulipotyphla, revealing that cross-family transmission predominantly occurs between shrews and moles. Moles may act as intermediaries in virus transmission among different shrew species, as we observed multiple cross-species transmitting viruses in both shrews and a mole species. In contrast, hedgehogs seem to occupy a more isolated role, harboring viruses that are more host-specific and showing a marked host tropism towards various hedgehog species.

Among shrew species, *S*. *murinus* appears to harbor more host-specific viruses, whereas *A*. *squamipes* and *Cr*. *dracula* carry viruses with greater cross-species transmission potential. Notably, the former two shrew species have been found to harbor human pathogens, highlighting the need for further investigation to comprehensively map the virome of these species and assess the potential risks of spillover.

Our study has a few limitations. Firstly, we pooled different lung tissues into a single sequencing library. This approach unfortunately precluded the observation of the viral distribution among different individuals. Secondly, there are still unclassified viral contigs that do not align with any known viral family. These enigmatic sequences hold the potential to represent novel viruses, but further research is essential to accurately identify and characterize them. Thirdly, certain rare species, including *So*. *cylindricauda*, *H*. *suillus*, *S*. *etruscus*, and *Cr*. *suaveolens* were not comprehensively represented in our study. Given their potential to impact the richness and abundance of our data, their under-representation could introduce biases. The difficulty of capturing these elusive insectivores poses a significant challenge. Finally, as datasets expand and information becomes increasingly complex, it is unlikely that we could include all relevant data, identified hosts, and phylogenetic details of viruses retrieved from NCBI.

There is no doubt that our findings hold significant value for surveillance purposes. We propose that certain identified viruses can be designated as target pathogens for future surveillance efforts. The genetic markers of these viruses can be used to develop specific diagnostic tools. These tools can then be effectively deployed in areas characterized by high human-wildlife interaction or in regions where the host species of these viruses are abundant. Moreover, this precisely aligns with our next-step plan, which is to conduct systematic epidemiological investigations and monitoring on animal individuals across a broader geographical area.

## Conclusions

In conclusion, this study identified a multitude of novel viruses in insectivores. It has unveiled intricate associations between the virome and risks, and substantially deepened our understanding of the extensive diversity of mammalian viruses carried by insectivores within a relatively restricted area. This understanding forms a vital foundation for the proactive prevention and control of emerging zoonotic pathogens. We firmly advocate for the establishment of a comprehensive and long-term multi-factor surveillance strategy, which focus on the continuous monitoring of the virome in insectivores and other potential reservoirs, with a particular emphasis on high-risk viruses, host species capable of cross–species transmission, and ecological factors. By doing so, we can take proactive steps to prevent potential zoonotic disease outbreaks and protect public health from emerging threats.

## Supplementary Information

Additional file 1: Fig S1. Phylogenetic trees of viruses, related to Fig. 4. Phylogenetic trees based on amino acid sequences of the RNA-dependent RNA polymerase (RdRp) protein for RNA viruses and the replicase protein for DNA viruses. Colored sequence names are viral sequences discovered in this study, and the blue, red, yellow colors represent host type of shrews, moles and hedgehogs respectively. Novel discovered viruses in this study are labeled with black rectangles, and viruses of known species are labeled with black circles.

Additional file 2: Fig S2. The Individual verification of detailed genome structures for 57 novel viral Species.

Additional file 3: Table S1. Sampling information of insectivores in 12 counties in Yunnan province. Table S2. Pooling of samples of insectivores captured in Yunnan province. Table S3. Summary of the viruses detected in this study. Table S4. Virus species demarcation criteria used in this study.

## Data Availability

The meta-transcriptomic sequencing reads generated in this study have been deposited in the SRA database (BioProject: PRJNA1160186, Private link, for editors and reviewers only: https://dataview.ncbi.nlm.nih.gov/object/PRJNA1160186?reviewer=secboifpvpi51ci60iqu1o38rv). The viral contig sequences generated in this study have been deposited in the NCBI GenBank database under accession numbers PQ421752 - PQ421863, PQ463690 and PQ463691 (Private link, for editors and reviewers only: https://figshare.com/s/94a945951000d4ba90ff).

## Abbreviations

RdRp: RNA-dependent RNA polymerase
EIDs: Emerging infectious diseases
ICTV: International Committee on Taxonomy of Viruses
RPM: Reads per million
PCA: Principal Component Analysis
PCoA: Principal coordinates analysis
NCBI: National Center for Biotechnology Information
SEOV: Orthohantavirus seoulense
MojV: Mojiang paramyxovirus
*H.*: *Hylomys*
*U.*: *Uropsilus*
*Cr.*: *Crocidura*
*Ep.*: *Episoriculus*
*So.*: *Sorex*
*S.*: *Suncus*
*A.*: *Anourosorex*
ds: Double-stranded
ss: Single-stranded
nt: Nucleotide
nr: Non-redundant
aa: Amino acid
