# Supplementary figures and images for: Host taxonomy and environment shapes insectivore viromes and viral spillover risks in Southwestern China
Source: Microbiome. 2025 May 16;13:122. doi: 10.1186/s40168-025-02115-9 (PMC12083107; doi:10.1186/s40168-025-02115-9)

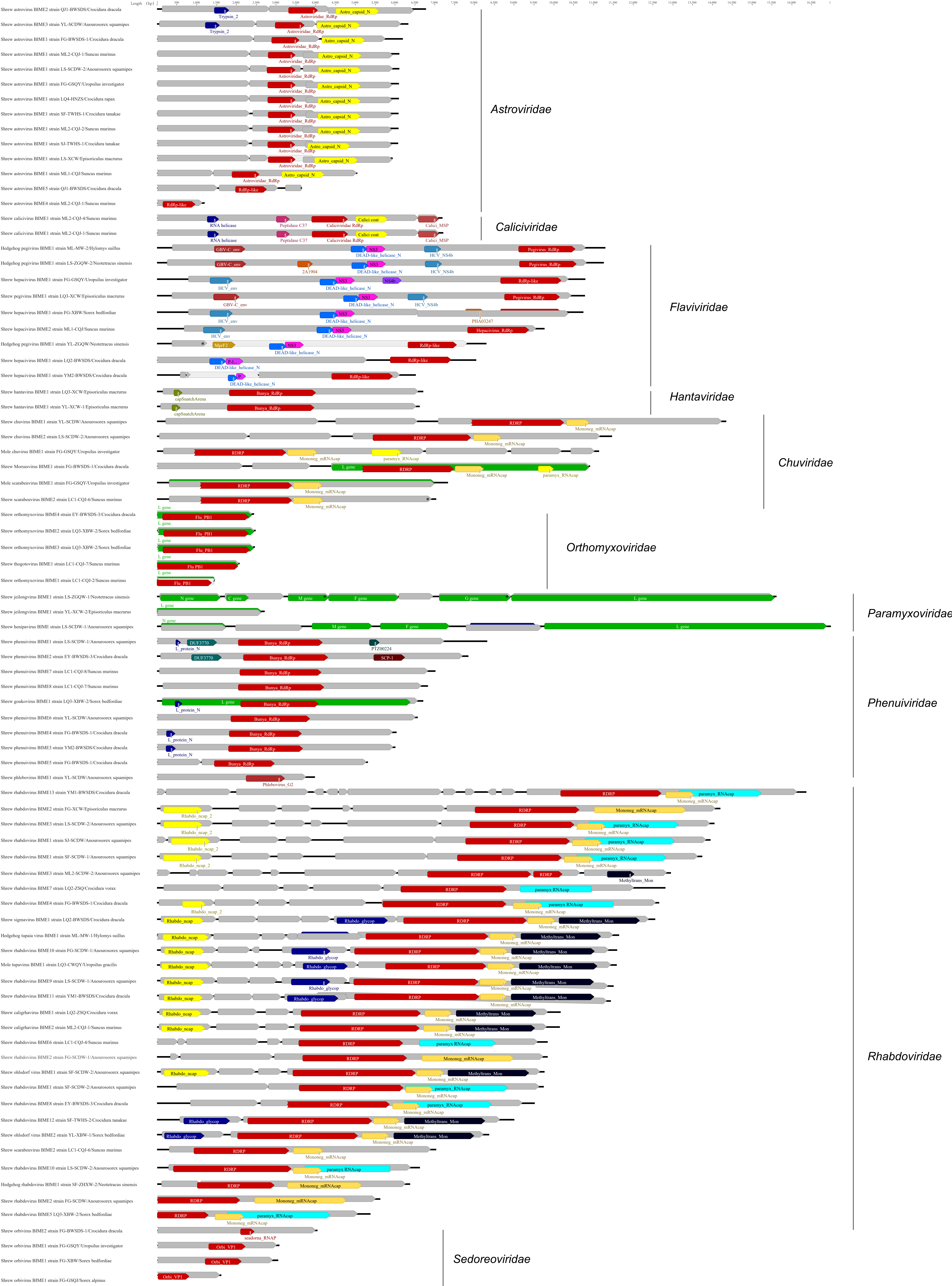

Supplement: Supplementary file 3 — Additional file 2: Fig S2. The Individual verification of detailed genome structures for 57 novel viral Species. [file 40168_2025_2115_MOESM2_ESM.pdf]
