# Supplementary material for: Host taxonomy and environment shapes insectivore viromes and viral spillover risks in Southwestern China
Source: Microbiome. 2025 May 16;13:122. doi: 10.1186/s40168-025-02115-9 (PMC12083107; doi:10.1186/s40168-025-02115-9)
Supplement: Supplementary file 2 — Additional file 1: Fig S1. Phylogenetic trees of viruses, related to Fig. 4. Phylogenetic trees based on amino acid sequences of the RNA-dependent RNA polymerase (RdRp) protein for RNA viruses and the replicase protein for DNA viruses. Colored sequence names are viral sequences discovered in this study, and the blue, red, yellow colors represent host type of shrews, moles and hedgehogs respectively. Novel discovered viruses in this study are labeled with black rectangles, and viruses of known species are labeled with black circles. [file 40168_2025_2115_MOESM1_ESM.pdf]

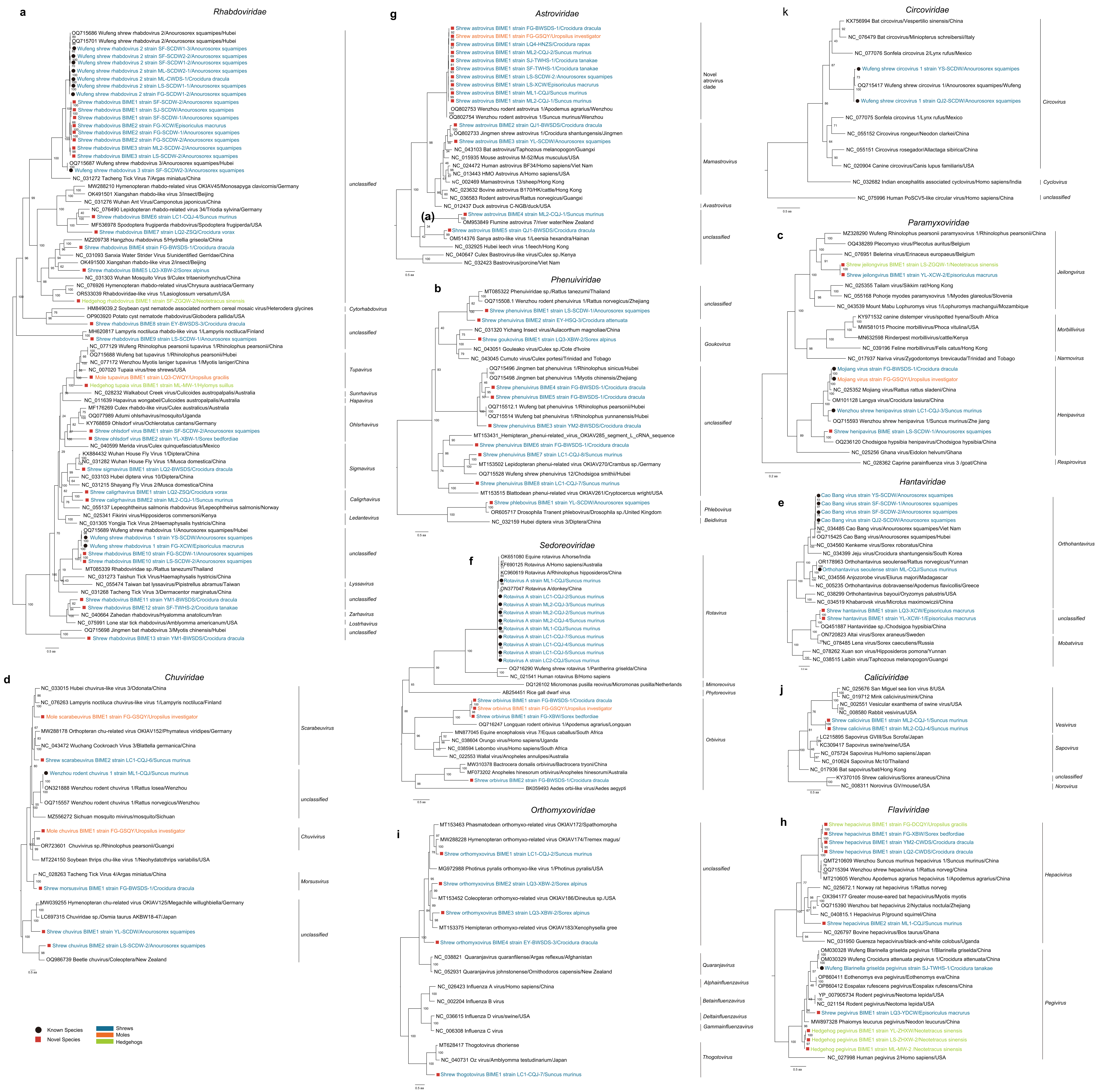

a
Rhabdoviridae
unclassified
Cytorhabdovirus
unclassified
Tupavirus
Sunrhavirus
Hapavirus
Ohlsrhavirus
Sigmavirus
Caligrhavirus
Ledantevirus
unclassified
Lyssavirus
unclassified
Zarhavirus
Lostrhavirus
unclassified
g
Astroviridae
Novel atrovirus clade
Mamastrovirus
Avastrovirus
unclassified
b
Phenuiviridae
unclassified
Goukovirus
unclassified
Phlebovirus
Beidivirus
f
Sedoreoviridae
Rotavirus
Mimoreovirus
Phytoreovirus
Orbivirus
i
Orthomyxoviridae
unclassified
Quaranjavirus
Alphainfluenzavirus
Betainfluenzavirus
Deltainfluenzavirus
Gammainfluenzavirus
Thogotovirus
k
Circoviridae
Circovirus
Cyclovirus
unclassified
c
Paramyxoviridae
Jeilongvirus
Morbillivirus
Narmovirus
Henipavirus
Respirovirus
e
Hantaviridae
Orthohantavirus
unclassified
Mobatvirus
j
Caliciviridae
Vesivirus
Sapovirus
unclassified
Norovirus
h
Flaviviridae
Hepacivirus
Pegivirus
d
Chuviridae
Scarabeuvirus
unclassified
Chuvivirus
Morsusvirus
unclassified
Known Species
Novel Species
Shrews
Moles
Hedgehogs
